# A comparative study of immediate versus delayed implant placement in periodontally compromised patients

**DOI:** 10.6026/973206300220508

**Published:** 2026-01-31

**Authors:** Geetika Kumar, Jitendra Kumar Diwakar, Ashutosh Agarwal, Manvi Chandra Agarwal, Prerna Agarwal

**Affiliations:** 1 Department of Periodontology and Implantology, Institute of Dental Sciences, Bareilly, Uttar Pradesh, India; 2 Department of Oral and Maxillofacial Surgery, Institute of Dental Sciences, Bareilly, Uttar Pradesh, India

**Keywords:** Bone preservation, dental implants, implant stability, periodontal disease, patient satisfaction

## Abstract

The optimal timing of dental implant placement in periodontally compromised patients remains controversial due to concerns regarding bone preservation and implant stability. Therefore, it is of interest to compare the clinical outcomes of immediate versus delayed dental implant placement in such patients. A total of 200 participants were randomly allocated to Group A (immediate placement) and Group B (delayed placement). Implant success rates, bone preservation, soft tissue health, implant stability, and patient satisfaction were evaluated. Although both groups showed high success rates, delayed implant placement demonstrated superior bone preservation, greater implant stability, and improved long-term functional outcomes.

## Background:

Dental implants have revolutionized the field of restorative dentistry, offering an effective solution for edentulous patients or those with compromised dentition [1]. However, in patients with periodontally compromised teeth, the timing of implant placement remains a topic of significant debate. The decision to place dental implants immediately after tooth extraction (immediate implant placement) or after a period of healing (delayed implant placement) is critical to the overall success and longevity of the implant, as well as the functional and aesthetic outcomes [2]. Periodontally compromised patients, typically characterized by advanced periodontal disease or bone loss, present unique challenges for implant placement. These patients often require a comprehensive approach, combining periodontal treatment with the careful planning of implant surgery to ensure successful outcomes [3]. The underlying goal of any implant treatment is to achieve osseointegration, which refers to the direct structural and functional connection between the bone and the implant surface. However, factors such as infection, bone quality and the degree of periodontal disease can significantly affect the healing process and the success of the implant [4]. Immediate implant placement, as the term suggests, involves placing the dental implant into the extraction socket immediately after tooth removal. The potential benefits of this approach include reduced treatment time, preservation of the bone and soft tissue structure and a more aesthetic outcome due to less tissue loss during the healing phase [5]. Furthermore, some studies suggest that immediate implant placement may help prevent resorption of the alveolar bone, a common issue in post-extraction scenarios. In contrast, delayed implant placement involves a waiting period between tooth extraction and implant placement, typically 3 to 6 months, to allow for the healing of the surrounding bone and soft tissue [6]. This approach aims to ensure the availability of adequate bone volume and better initial stability for the implant. However, the controversy lies in the comparison of the long-term effectiveness and predictability of both approaches. Some studies suggest that immediate implant placement in periodontally compromised patients may lead to an increased risk of complications, such as implant failure due to inadequate osseointegration, infection, or bone loss [7]. On the other hand, delayed implant placement is thought to offer more controlled healing conditions, but it may involve the risk of additional bone resorption during the waiting period, which can necessitate bone grafting procedures and extend the treatment timeline [8]. The clinical outcomes of these two techniques include implant survival rates, soft tissue and bone levels, functional outcomes and patient satisfaction, need thorough investigation. Additionally, factors like the patient's general health, the presence of systemic conditions, oral hygiene and the degree of periodontal compromise all play a role in determining the optimal treatment plan [9]. Therefore, it is of interest to determine the most effective and predictable approach for implant placement in periodontally compromised patients, weighing the benefits and risks of both immediate and delayed implant placement techniques.

## Methodology:

This study aimed to compare the outcomes of immediate versus delayed implant placement in periodontally compromised patients. A total of 200 participants were enrolled, who met the eligibility criteria for the study. The methodology involved a randomized controlled trial (RCT) design to ensure an unbiased comparison between the two treatment approaches. The inclusion criteria for participants included adults aged 18 to 70 years with periodontally compromised teeth requiring extraction due to Grade II or III mobility or severe periodontal disease. Participants were also required to have sufficient bone volume for implant placement, as determined by pre-operative radiographic assessment. Additionally, they were required to have no history of systemic diseases that could affect bone healing (such as uncontrolled diabetes or autoimmune disorders) and must have been willing to maintain good oral hygiene throughout the study period. Exclusion criteria included patients with contraindications for implant surgery, insufficient bone height or width in the extraction site, smokers exceeding 10 cigarettes per day and those with a history of radiation therapy or bisphosphonate use. The study utilized a RCT design, where participants were randomly assigned to one of two groups: the Immediate Implant Placement Group (Group A) and the Delayed Implant Placement Group (Group B). Group A underwent implant placement immediately after tooth extraction, while Group B underwent implant placement after a healing period of 3 to 6 months following tooth extraction. Randomization was done using a computer- generated sequence to minimize bias in group allocation. Before surgery, participants underwent a comprehensive pre-surgical assessment, which included a detailed medical history to screen for any contraindications. Radiographic assessments, including cone beam computed tomography (CBCT), were performed to evaluate bone volume, bone density and any relevant anatomical structures in the region of the planned implant placement. Clinical periodontal measurements, such as probing depth, attachment level and gingival recession, were also recorded at baseline. The surgical procedure differed between the two groups. In Group A, implants were placed into the fresh extraction sockets immediately after tooth removal. All participants received the same type and size of implant. In Group B, the extraction sites were allowed to heal for 3 to 6 months before implant placement, during which any necessary procedures like bone grafting or soft tissue management were performed as required. Post-operative care followed a standardized protocol for all participants, including prescribed antibiotics and analgesics, along with instructions for post-surgical care. Follow-up visits were scheduled at 1 week, 1 month, 3 months and 6 months after surgery to monitor healing and assess any complications. The primary outcomes of the study included implant success rates, defined as the absence of implant failure, infection, or mobility. Bone levels around the implant were measured using radiographic analysis, with a focus on crestal bone loss at 6 months. Soft tissue health was also assessed by measuring gingival inflammation, recession and papillary height at 6 months post-surgery. Functional outcomes included implant stability, measured using resonance frequency analysis and the ability to successfully restore the implant. Secondary outcomes focused on patient satisfaction, assessed using a Visual Analog Scale (VAS) to measure pain, discomfort and aesthetic outcomes. Healing time was recorded; particularly focusing on the time required for soft tissue healing and implants osseointegration. Statistical analysis was performed using SPSS software. Descriptive statistics, such as means, standard deviations and frequencies, were used to summarize the data. The Chi-square test was applied to categorical variables, such as implant failure, while the independent t-test was used for continuous variables, such as bone loss and healing time. A p-value of <0.05 was considered statistically significant. The study adhered to ethical guidelines outlined in the Declaration of Helsinki and was approved by the Institutional Review Board (IRB). Written informed consent was obtained from all participants, ensuring they understood the nature of the study and any potential risks involved.

## Results:

The results of this study compared the clinical outcomes of immediate versus delayed implant placement in periodontally compromised patients. A total of 200 participants were enrolled and completed the study, with 100 participants in each group. The primary outcomes measured were implant success rates, bone loss, soft tissue health and implant stability. Secondary outcomes included patient satisfaction and healing time. The implant success rate was calculated based on the absence of implant failure, infection, or mobility. The results revealed that both groups had high implant success rates, but there was a significant difference between the two groups. Group A (Immediate Implant Placement) showed a slightly lower success rate compared to Group B (Delayed Implant Placement) (Table 1). Crestal bone loss was measured at 6 months post-surgery. The results indicated that the bone loss in the immediate implant placement group was significantly higher compared to the delayed implant placement group, suggesting better bone preservation in the delayed placement group (Table 2). Soft tissue health was evaluated by measuring gingival inflammation, recession and papillary height at 6 months. The immediate implant placement group exhibited slightly higher levels of gingival recession and inflammation compared to the delayed implant placement group, although the differences were not statistically significant (Table 3). Implant stability was measured using RFA at 6 months post-surgery. The results showed that Group B (Delayed Implant Placement) had significantly higher stability values compared to Group A (Immediate Implant Placement), suggesting better initial implant stability after a healing period (Table 4). Patient satisfaction was assessed using VAS to measure pain, discomfort and aesthetic outcomes at 6 months post-surgery. Group B (Delayed Implant Placement) had higher satisfaction scores, particularly regarding pain and aesthetic outcomes, while Group A (Immediate Implant Placement) had slightly higher discomfort levels during the healing period. The healing time, defined as the time taken for soft tissue healing and osseointegration, was significantly shorter for Group A (Immediate Implant Placement) compared to Group B (Delayed Implant Placement). However, Group B had more stable and favorable long-term outcomes (Table 5). All data were analyzed using SPSS software. The Chi-square test was applied to compare implant success rates between the groups and the independent t-test was used to compare continuous variables such as bone loss, implant stability and patient satisfaction. A p-value of <0.05 was considered statistically significant.

## Discussion:

This study compared the clinical outcomes of immediate versus delayed implant placement in periodontally compromised patients, with a total of 200 participants. The findings of this study showed that while both techniques had high implant success rates, delayed implant placement resulted in better long-term outcomes, particularly in terms of bone preservation, implant stability and patient satisfaction. These results are consistent with the existing literature but also highlight some critical nuances that can influence clinical decision- making. The implant success rates observed in this study were 92% for the immediate implant placement group (Group A) and 98% for the delayed implant placement group (Group B). The slightly higher success rate in the delayed implant group aligns with Patel *et al.* (2023) [10], who reported a similar trend in their meta-analysis, where delayed placement generally resulted in a marginally higher survival rate compared to immediate placement. However, both approaches had high success rates, indicating that both can be viable treatment options when performed under the right conditions. This finding suggests that although immediate implant placement is often chosen for its convenience and shorter treatment timeline, delayed placement may provide better long-term results, particularly in periodontally compromised patients who require optimal bone and tissue healing. One of the most significant findings of this study was the bone loss measurement. At 6 months post-surgery, the immediate implant placement group exhibited more significant bone loss (mean of 2.1 mm) compared to the delayed group (mean of 1.2 mm). This is consistent with Singh *et al.* (2021) [11], who reported more crestal bone loss in immediate implants at both 3 and 6 months. Bone resorption is a common issue following immediate implant placement, especially in compromised bone sites and can negatively affect the long-term success of the implant. This study supports the idea that delaying implant placement allows time for bone remodeling and healing, leading to better preservation of the alveolar bone and potentially improving the outcomes of implant surgery. In terms of soft tissue health, the results showed that the immediate implant placement group had slightly more gingival recession and inflammation compared to the delayed placement group, although the differences were not statistically significant. This aligns with the findings of Garcia-Sanchez *et al.* (2022) [12], who found that immediate implants often result in more early complications, including soft tissue recession, due to the lack of time for the tissues to heal properly before implant placement. However, despite these early complications, immediate implants can be beneficial in cases where esthetics and reduced treatment time are prioritized, as seen in other studies. Implant stability was another important outcome in this study, with delayed implants showing higher resonance RFA scores, indicating better initial stability. Schiegnitz *et al.* (2024) [13] similarly found that delayed implant placement provided better implant stability, particularly in patients with compromised periodontal conditions. The delayed placement allows for proper bone healing, resulting in improved primary stability and, therefore, better long-term outcomes. Regarding patient satisfaction, the delayed implant placement group reported higher satisfaction scores, particularly concerning pain and aesthetic outcomes. This result aligns with findings from recent studies that suggest patients who undergo delayed implant placement experience less discomfort during the healing phase and report better aesthetic results in the long term [14]. The immediate implant placement group, while benefiting from a faster treatment timeline, had slightly more discomfort and a lower level of satisfaction with the aesthetic outcomes. The healing time was significantly shorter for the immediate implant group (mean of 3 months) compared to the delayed group (mean of 5.4 months). This finding is consistent with other clinical reports that show immediate implants can offer a shorter overall treatment time, which is a key advantage in certain clinical settings (Cosyn, 2020). However, the delayed implant group had more stable long-term outcomes, suggesting that faster healing does not always equate to better overall success.

## Conclusion:

We show that both immediate and delayed implant placement techniques in periodontally compromised patients result in high success rates. However, delayed implant placement exhibited superior long-term outcomes, particularly in bone preservation, implant stability and patient satisfaction. These findings suggest that while immediate placement offers quicker results, delayed placement may provide more predictable, stable results in the long run. Therefore, careful consideration of the patient's specific condition is essential for optimizing treatment planning in periodontal cases.

## Figures and Tables

**Table 1:** Implant success rate comparison

**Group**	**Success Rate (%)**	**Failure Rate (%)**
Immediate Implant Placement (Group A)	92	8
Delayed Implant Placement (Group B)	98	2

**Table 2:** Bone loss at 6 months post-surgery

**Group**	**Mean Bone Loss (mm)**	**Standard Deviation**
Immediate Implant Placement (Group A)	2.1	0.8
Delayed Implant Placement (Group B)	1.2	0.6

**Table 3:** Soft Tissue health at 6 months

**Group**	**Gingival Recession (mm)**	**Gingival Inflammation (VAS)**	**Papillary Height (mm)**
Immediate Implant Placement (Group A)	1.4	4.3	3.2
Delayed Implant Placement (Group B)	1	3.7	3.5

**Table 4:** Implant stability (RFA Scores)

**Group**	**Mean RFA Score**	**Standard Deviation**
Immediate Implant Placement (Group A)	70.4	5.2
Delayed Implant Placement (Group B)	74.6	4.8

**Table 5:** Healing time comparison

**Group**	**Mean Healing Time (Months)**	**Standard Deviation**
Immediate Implant Placement (Group A)	3	0.5
Delayed Implant Placement (Group B)	5.4	0.8
